# Well-being, character strengths, and depression in emerging adults

**DOI:** 10.3389/fpsyg.2023.1238105

**Published:** 2023-09-20

**Authors:** Almir Diego Brito, Adriana B. Soares

**Affiliations:** ^1^Department of Psychology, Salgado de Oliveira University, Niterói, Brazil; ^2^Department of Psychology, Rio de Janeiro State University, Rio de Janeiro, Brazil

**Keywords:** emerging adulthood, depression, character strengths, positive psychology, well-being

## Abstract

**Introduction:**

Emerging adulthood is a period of transition between adolescence and adulthood, during which individuals explore different roles and possibilities. This period is often characterized by high levels of stress and uncertainty, which can increase the risk of depression. The aim of this study was to investigate the associations between emerging adulthood dimensions, well-being, character strengths and depression, as well as to verify the differences in the levels of these elements among different groups of depressive symptomatology.

**Methods:**

Participants were 1,060 individuals (741 females, 305 males) between the ages of 18 and 30 years (*M* = 22.3, SD = 3.72). Spearman’s correlation, linear regression, and Kruskal- Wallis with pairwise *post-hoc* analyses were used to analyze the data.

**Results:**

There were significant positive correlations between emerging adulthood dimensions, well-being, and character strengths, and negative correlations between these variables and depression. There were also significant differences in the means of the evaluated constructs among the symptomm groups.

**Discussion:**

The findings suggest that emerging adulthood dimensions, well-being, and character strengths are all associated with depression in emerging adults and have important implications for the provision of interventions in health and education for emerging adults. The study provides a deeper understanding of the factors influencing depression during emerging adulthood and emphasize the importance of promoting positive psychology elements and developing personalized interventions for emerging adults. This also suggest that public policies related to mental health and education should take into account the unique needs of emerging adults.

## 1. Introduction

Depression is a mental disorder with symptoms such as sadness, loss of pleasure and feelings of worthlessness (American Psychiatric Association, 2013). Its etiology is complex and involves emotional, cognitive, biological and contextual factors (Tehranchi et al., 2018). The prevalence of depression is high during the transition to adulthood, however, the provision of specialist care is low (Arnett, 2018). People with low levels of positive psychological attributes, such as character strengths and well-being, may be more likely to experience depression (Tehranchi et al., 2018). This study investigated the associations between these constructs and differences in emerging adults with different levels of depressive symptomatology. It is intended that its results can support care policies for emerging adults, such as, for example, the provision of Interventions in Positive Psychology.

### 1.1. Emerging adulthood and mental health

Traditional transition into adulthood has changed in the early 21st century. Emerging adulthood is a new model of human development between the ages of 18 and 30, characterized by exploratory experiences related to career and affective relationships, including the postponement of marriage and parenthood (Arnett, 2018). It is a favorable period for the exploration of identity (1), especially in academic, professional and affective areas. Many options can generate instability (2) due to the fluidity of choices. Frequent changes and the lack of definition of roles generate ambivalence (3), causing the self-concept to oscillate between adolescence and adult life. In general, emerging adults feel the need to establish their lives, carry out their projects, which can lead to a greater focus on themselves (4) and states of optimism (5). These last elements favor decision-making and self-realization (Sánchez-Queija et al., 2020).

Emerging adulthood can have negative implications for mental health. Identity exploration can lead to confusion and fear, especially for those with low self-efficacy. Instability can be aggravated by voluntary or involuntary changes. Some individuals may find it difficult to stay focused on themselves without social support. Ambivalence can lead to anxiety and depression. Optimism can help in facing difficult situations, but a lack of confidence can increase the risk of depression and anxiety (Arnett et al., 2014).

Depression, as a problem of a psychological nature, often affects emerging adults; however, most of these individuals do not receive treatment for this disease or resources for its prevention (Kia-Keating et al., 2017). The stressors present in this phase, such as dealing with academic, financial and social difficulties, can negatively alter their mental health, possibly causing depression, depending on the individual’s personal and contextual characteristics (Mahmoud et al., 2012). Emerging adulthood is a high-risk period for depression. Emerging adults with depression are more likely to experience relationship problems, substance abuse, and academic setbacks (Kuwabara et al., 2007).

The social institutions (family, government, education, health, and labor market) have not been able to adequately address the unique needs of this stage. This lack of provision of appropriate care stands in contrast to the support that is typically provided during earlier stages of development, where parents often take their children for various treatments, including mental health, and educational and health approaches are tailored to meet the specific needs of childhood and adolescence (Arnett et al., 2014).

Tanner (2016) argues that the transition to adulthood should be seen as a period of mental health care. It is important to focus on individual needs and avoid comparing oneself to others, especially in terms of educational and professional achievements. Baggio et al. (2017) found that emerging adults are at risk for psychiatric disorders. They recommend assessing the psychological states of emerging adults in association with the five classic markers of this age proposed by Arnett (2000). Emerging adults with negative psychological states are less likely to have clarity about their future, while those with higher levels of psychosocial well-being are more optimistic about their ability to achieve their life goals (Baggio et al., 2017).

No other studies, other than that of Baggio et al. (2017) were found in the literature that related markers of emerging adulthood with depression. However, other investigations sought to investigate some risk and protective factors among the emerging adult population. Cardoso et al. (2018) found negative affect, anxiety, and depression in individuals diagnosed with depression. Pereira et al. (2018) found that individuals without experience of suicidal ideation had higher levels of self-efficacy, self-esteem and family relationships, while the others had higher levels of social anxiety. The study by Dutra-Thomé et al. (2017) highlighted the importance of personal resources, family and educational support in overcoming difficulties. Cohen et al. (2019) found that prior depression, abuse, and exposure to neglect increased the risk of current depression in emerging adults. The study by O’Connor et al. (2017) highlighted that positive mental health in adolescence was associated with educational achievements, career success, and civic responsibility in adulthood. The study by Othman and Jaafar (2022) found no significant correlation between the elements of emerging adulthood and depression, however, there were positive predictions of negativity and of a self-focus on depression.

In addition to the common challenges at this stage of life that negatively affect the mental health of emerging adults, the COVID-19 pandemic, a disease caused by the SARS-CoV-2 virus, which started in 2020, caused even more negative consequences and limitations in their development and well-being. A study of 1,200 american university students found that one-third reported suicidal ideation and two-thirds experienced symptoms of severe anxiety and depression (Halliburton et al., 2021). Another study of 98 emerging adults found that the pandemic caused a significant increase in mental health problems in participants who already had some pre-existing psychopathology (Van den Berg et al., 2021). Maternal support was associated with decreased psychological distress and depressive symptoms, while paternal support was associated with increased psychological distress and depressive symptoms. A Portuguese study by Maia and Dias (2020) of university students found that levels of depression, anxiety and stress were significantly higher during the pandemic than before the pandemic. These findings highlight the importance of mental health care for emerging adults during times of crisis.

### 1.2. Emerging adulthood and positive psychology

Although many emerging adults are exposed to adverse and stressful situations that can hinder their development, especially those who are economically disadvantaged, Lo-oh (2019) argued that there has been an increase in the amount of evidence related to this stage as a period of positive development. The author highlighted that the theoretical and practical field of Positive Psychology enables a new perspective on the emotional and social development of emerging adults, transferring attention from risks to studies that value positive personal resources.

Two important contributors to positive development and protection against depression in emerging adults are well-being according to the PERMA model (Seligman, 2012) and character strengths (Peterson and Seligman, 2004). From these two sets of psychosocial elements, it is possible to understand the positive development of emerging adults approaching the two blocks proposed by Padilla-Walker and Nelson (2017), considering, therefore, the positive personal characteristics (character strengths) and dimensionally measuring well-being (PERMA model).

Peterson and Seligman’s (2004) definition of character strengths corresponds to a set of stable traits of an individual’s personality strengths that contributes to a good life for themself and others. Based on scientific, social and philosophical arguments, they proposed a model called the Values in Action (VIA) Classification of Strengths composed of 24 character strengths that are divided into six virtues: (1) wisdom and knowledge (creativity, curiosity, open mindedness, love of learning, perspective); (2) courage (bravery, perseverance, honesty, zest); (3) humanity (love, kindness, social intelligence); (4) justice (teamwork, fairness, leadership); (5) temperance (forgiveness, modesty, prudence, self-regulation) and (6) transcendence (appreciation of beauty, gratitude, hope, humor, spirituality). Despite being found in factorial measurement instruments, they should be considered more as a list of positive traits that have been empirically shown to contribute to a positive life, rather than just a psychological measure (Wagner et al., 2019).

The PERMA model proposed by Seligman (2012) is composed of five factors that determine and measure well-being, namely: (1) positive emotions (experience of positive affects); (2) engagement (state of focus and experience of flow in activities) (3) relationships (experience of positive interpersonal relationships); (4) meaning (sense of purpose in life) and (5) accomplishments (setting and achieving goals and objectives). The initials of these components (Positive emotions, Engagement, Relationships, Meaning, Accomplishments) form the acronym PERMA. From this perspective, it is possible to consider well-being in a multidimensional way, which is fundamental for a more adequate understanding of positive psychological functioning (Butler and Kern, 2016).

Studies such as those by Gander et al. (2016), Wąsowicz et al. (2021), Ryan et al. (2019) and Kunjiapu and Kunasegaran (2021) found moderate negative associations between the PERMA factors and depression, while Kern et al. (2015) and Falecki et al. (2018) reported negative predictive relationships. Frye and Liem (2011) and Salmela-Aro et al. (2008) found a higher prevalence of depressive symptoms in groups with lower and moderate levels, respectively. Wagner et al. (2019) and Littman-Ovadia et al. (2017) found positive associations between character strengths and the PERMA dimensions, as well as between experiences of character strengths and experiencing positive emotions, satisfying relationships, and greater professional and academic engagement.

There are still few studies that have simultaneously investigated character strengths and PERMA well-being among emerging adults. The work of Leontopoulou and Triliva (2012) with 516 Greek university students between 18 and 29 years of age stands out, in which the prediction of the five PERMA factors and four character strengths (curiosity, gratitude, love of learning and humor) on another three well-being indicators (flourishing, resilience and positive perception) was analyzed.The three dependent variables were predicted by the PERMA factors and the four character strengths. In view of the results, the authors emphasized that the multidimensionality of the PERMA allows greater specificity in identifying and addressing the needs of emerging adults, which can be systematically assisted through actions aimed at their positive development.

There are only a small number of studies that have investigated the character strengths of emerging adults. Of these studies, only Noftle (2015) and Craig et al. (2021) evaluated all 24 character strengths in this population, while the others focused on only some of them (Leontopoulou and Triliva, 2012; Leontopoulou, 2015; Gomez-Baya et al., 2021; Kadir and Mohd, 2021). Furthermore, none of these studies explored the relationship between character strengths and depression. Due to the scarcity of studies that have related character strengths to depression in emerging adulthood, it is necessary to consider investigations with other age groups to advance the discussion of the results. This is the case of reports by Tehranchi et al. (2018) who selected seven character strengths (hope, critical thinking, emotional intelligence, gratitude, forgiveness, spirituality and zest) to assess the etiology of depressive symptoms in a sample of 200 Iranians between 18 and 50 years of age. The results demonstrated that these forces influenced the direct relationships of dysfunctional attitudes and happiness with depression. That is, the seven forces had an indirect effect on depression.

Duan and Wang (2018) found in a sample of 3,536 Chinese that people found that those with lower levels of character strengths were more likely to be depressed. Another study of 1,411 Iranian adolescents found that those with lower levels of character strengths were more likely to be depressed (Jabbari et al., 2021). Casali et al. (2021) presented a study of 944 Italians found that those with lower levels of hope, enthusiasm, and love were more likely to be depressed. According to Anjum and Amjad (2016), university students who received counseling had higher scores for depression, followed by those who needed counseling. The students receiving clinical care scored lower in all character strengths.

### 1.3. The present study

Considering the significant impact of depression during emerging adulthood, as emphasized by Kuwabara et al. (2007), and the limited scientific and institutional approaches (in health, education, and workplaces) compared to other stages of development such as childhood and old age, previous studies have sought to identify the risk and protective factors for depression in emerging adults. Many of these studies have focused on investigating factors such as personal history, sociodemographic status, education, race, previous traumatic experiences, physical health, and substance use (Frye and Liem, 2011; Mondi et al., 2017; Cohen et al., 2019; Germani et al., 2020; Alzueta et al., 2021). Despite highlighting the problem of the prevalence of this disease for the healthy development of this population, these studies did not address the positive psychological resources associated with depression in emerging adulthood, as proposed here. Other investigations have indicated that young people with a more optimistic tendency toward the future (Klimusová et al., 2016) and with higher self-esteem (Moore II and Shell, 2017; Germani et al., 2020) have healthier behaviors and are less likely to develop depression, thus reinforcing that psychosocial elements affect the development of this disorder.

Given that the presence of Positive Psychological elements is associated with better mental health, the current study has three main goals. First, it was evaluated the associations between emerging adulthood, well-being, character strengths and depression. The research question is whether there is a significant relationship between these constructs among individuals in the emerging adulthood stage. It is hypothesized that the elements of well-being and character strengths remain negatively associated with depression, with the exception of the negativity and feeling in between elements of emerging adulthood, which should be negatively correlated with depression (Casali et al., 2021; Kunjiapu and Kunasegaran, 2021).

Secondly, it was verified the prediction of emerging adulthood, well-being, character strengths regarding the depression disorder. The research question is whether emerging adulthood, well-being, and character strengths can significantly predict the presence or absence of depression. This second hypothesis postulates is that higher levels of well-being, character strengths and emerging adulthood dimensions adulthood (with the exception of negativity and feeling in between elements) prevent a lower likelihood of suffering from depression (Falecki et al., 2018; Tehranchi et al., 2018; Othman and Jaafar, 2022).

Third, it was compared the variations in the levels of emerging adulthood well-being, character strengths in different depression symptomatological groups. The research question is whether there are significant differences in these constructs among individuals with varying levels of depression symptoms. The research question is whether there are significant differences in these constructs between individuals with varying levels of depression symptoms. This third hypothesis suggests that individuals with lower depression symptoms exhibit higher levels of emerging adulthood (with the exception of negativity and feeling in between elements), well-being and character strengths compared to those with higher depression symptoms (Frye and Liem, 2011; Anjum and Amjad 2016).

## 2. Methods

### 2.1. Participants

Participants were 1,060 emerging adults, aged between 18 and 30 years (*M* = 22.3; *SD* = 3.72), 741 women (70%), 305 men (29%) and 14 of other genders (1%). Income was classified according to the following indicators: class A (*n* = 87) (8%), class B1 (*n* = 135) (13%), class B2 (*n* = 361) (35%), class C1 (*n* = 288) (28%), class C2 (*n* = 136) (13%) and class D/E (*n* = 27) (3%). The participants were divided into the following groups according to education: Incomplete Elementary Education (*n* = 2), Complete Elementary Education and Incomplete High School (*n* = 51), Complete High School and Incomplete Higher Education (*n* = 687), Complete Higher Education and Incomplete Master’s degree (*n* = 294), Complete Master’s degree and Incomplete doctorate (*n* = 26), and Complete doctorate (*n* = 1). Regarding employment, 87 did not work or study, 378 only studied, 249 only worked and 347 worked and studied. The types of residence were classified as follows: with friends/shared household/student housing (*n* = 55), with partner (*n* = 141), with parents or family members (*n* = 776) and alone (*n* = 89). Although this sociodemographic information presents different characteristics among the participants, there were no statistically significant differences between these data.

### 2.2. Instruments

The measurement instruments used in this study are presented below. It is worth emphasizing that the Cronbach’s alphas highlighted in parentheses refer to the data from the sample of this study.

Emerging Adulthood – Brazilian Version of the Inventory of the Dimensions of Emerging Adulthood (Dutra-Thomé et al., 2017). This self-report instrument consists of 29 items (e.g., “Time of focusing on yourself?”), divided into six factors, however only five were used: (1) Identity Exploration (five items, *α* = 0.72); (2) Possibilities (four items, *α* = 0.66); (3) Instability (seven items, *α* = 0.80); (4) Self-Focused (eight items, *α* = 0.74); (5) Feeling “In-Between” (four items, *α* = 0.71). Likert-type scale responses vary between four levels ranging from strongly disagree to strongly agree.

The PERMA well-being – Brazilian version of the PERMA-Profiler Flourishing Scale (Carvalho et al., 2023). This self-report instrument consists of 23 items (e.g., “In general, how often do you feel positive?”), three items for each PERMA dimension and three items about physical health, three items about negative emotions, one item about loneliness and one item that assesses happiness in general. Only the five factors referring to the PERMA were used in the study, with them presenting the following Cronbach’s alpha values: (1) positive emotions: *α* = 0.90; (2) engagement: *α* = 0.63; (3) relationships: *α* = 0.79; (4) meaning: *α* = 0.83; and (5) accomplishments: *α* = 0.79. The response scale has 11 Likert-type options ranging from never to always.

Character Strengths – Character Strengths Scale (Noronha and Barbosa, 2016). This self-report instrument has 71 items (e.g., “I think of different possibilities when I make a decision.”), three for each of the 24 character strengths, with the exception of Appreciation of beauty, which is composed of two items. Its structure is unidimensional with Cronbach’s alpha of 0.95. The Likert-type response scale has five options ranging from nothing to do with me to everything to do with me.

Depression – Depression, Anxiety and Stress Scale (DASS-21) (Martins et al., 2019). This self-report instrument has 21 items, however, only the 7-item depression scale was used (e.g., “I could not experience any positive feelings.”): (1) depression: *α* = 0.92. Responses are given on a four-point Likert-type scale ranging from Did not apply to me at all to Applied to me very much or most of the time.

### 2.3. Ethical procedures

The research project was submitted to the University Ethics Committee and approved. Before each application of the instruments, the participants signed the consent form.

### 2.4. Data collection procedures

The SurveyMonkey platform was used for data collection. The questionnaire was disseminated in groups of students from private and public universities, as well as groups of professionals and other discussions groups for young people on Facebook, WhatsApp and Twitter. Participants were also invited to share the questionnaire link with friends who were in the age range of the participants.

### 2.5. Data analysis procedures

The data collected by the platform were grouped in a database of the IBM SPSS STATISTICS 25 statistical analysis program. Descriptive analyses of the sample were performed, followed by parametric tests that indicated the non-normality of the sample. After this verification, Spearman’s Correlation was performed to verify the relationships between the constructs. The Multiple Linear Regression using the forward method was performed in order to identify which were the predictors of depression among the constructs emerging adulthood, PERMA well-being and character strengths. The Kruskal-Wallis test was performed to verify the differences between the averages of emerging adulthood, PERMA well-being and character strengths in the face of different levels of depression, followed by the post-hoc pairwise test.

## 3. Results

The prevalence of depression found in the participants made it possible to divide them into three groups that describe the levels of symptoms in these individuals. The symptomatological levels of depression proposed by Lovibond and Lovibond (1995) are divided into five groups: (a) acceptable = 0–9 points; (b) mild = 10–12 points; (c) moderate = 13–20 points; (d) severe = 21–17; and (e) extremely severe = 28–42 points. In order to make the understanding of the extensive results more parsimonious, it was decided to group acceptable with mild levels and severe with extremely severe levels, considering, after this division, three groups of symptomatic levels. Table 1 presents this classification in the groups called “acceptable/mild,” “moderate” and “severe/extremely severe.”

**Table 1:** Depression levels among participants.

Acceptable/Mild	Moderate	Severe/extremely severe
368 (34.7%)	228 (21.5%)	465 (43.8%)

Table 2 presents the results of the Spearman correlations of the factors of emerging adulthood, PERMA and character strengths with depression. Only the emerging adulthood factors of identity and bravery, and the kindness/generosity, modesty and prudence character strengths did not show significant correlations with depression.

**Table 2:** Spearman correlations of the factors of emerging adulthood, PERMA and character strengths with depression.

Variables	Factors	Depression
Emerging adulthood	Possibilities	−0.19^**^
	Identity	0.00
	Self-focused	−0.15^**^
	Negativity	0.38^**^
	Feeling “in between”	0.10^**^
PERMA	Positive emotions	−0.66^**^
	Engagement	−0.41^**^
	Relationships	−0.45^**^
	Meaning	−0.52^**^
	Accomplishments	−0.48^**^
Character strengths	Creativity	−0.14^**^
	Curiosity	−0.16^**^
	Open mindedness/openness to new ideas	−0.06^*^
	Love of learning	−0.27^**^
	Perspective/wisdom	−0.10^**^
	Honesty	−0.10^**^
	Bravery	−0.02
	Persistence	−0.32^**^
	Enthusiasm/zest	−0.58^**^
	Kindness/generosity	−0.05
	Love	−0.27^**^
	Social and emotional intelligence	−0.06^*^
	Justice	−0.06^*^
	Leadership	−0.22^**^
	Teamwork	−0.19^**^
	Forgiveness	−0.11^**^
	Humility	0.02
	Prudence	−0.03
	Self-regulation	−0.15^**^
	Appreciation	−0.19^**^
	Gratitude	−0.36^**^
	Hope and optimism	−0.44^**^
	Humor	−0.26^**^
	Spirituality	−0.28^**^

*p < 0.05; **p < 0.01.

A Multiple Regression Analysis (Forward method) was carried out with the aim of investigating the extent to which the emerging adulthood, PERMA and character strength factors had an impact on depression levels. The results showed that there was a significant influence of positive emotions, enthusiasm/zest, kindness/generosity, social/emotional intelligence, negativity, meaning, bravery and identity on depression [*F* (9.1051 = 123.885, *p* < 0.05), adjusted *R^2^* = 0.511]. Table 3 presents the coefficients for all significant predictors. As can be seen, the variable that was most strongly significant was positive emotions, explaining 42.7% of the outcome. The other factors, in turn, were related to only 8.8% of the depression variance. The other factors that did not present significance were not highlighted in Table 3.

**Table 3:** Prediction of emerging adulthood factors, PERMA characteristics, and character strengths on depression.

Predictors	Standardized coefficients (Beta)	*t*	Sig.	*R^2^*	*ΔR^2^*
Constant	-	7.58	0.00	-	-
Positive emotions	−0.35	−9.03	0.00	0.43	-
Enthusiasm/zest	−0.28	−8.07	0.00	0.45	0.44
Kindness/generosity	0.09	3.22	0.00	0.47	0.47
Social/emotional intelligence	0.13	5.18	0.00	0.48	0.48
Negativity	0.09	3.76	0.00	0.50	0.49
Relationships	−0.10	−3.64	0.00	0.51	0.50
Meaning	−0.11	−3.32	0.00	0.51	0.51
Bravery	0.08	2.95	0.00	0.51	0.51
Identity	0.05	2.28	0.02	0.52	0.51

*p < 0.05; **p < 0.01.

To verify the difference in means between groups, the Kruskal-Wallis test was performed, considering that the results of the Kolmogorov–Smirnov and Shapiro–Wilk normality tests did not indicate normality patterns for the sample data. Tables 4–6, respectively, show the data referring to the means, the results of the Kruskal-Wallis test and the pairwise post-hoc test for each factor of emerging adulthood, PERMA and character strengths in relation to the groups of depression symptoms.

**Table 4:** Means, Kruskal-Wallis and Post-hoc pairwise of emerging adulthood.

EA factors	Groups	Mean	Median	Standard deviation	Kruskal-Wallis			Post-hoc pairwise				
					H (2)	*p*	Rank	Pair	*p*	*z*	*r*	Effect
Possibilities	1	10.32	11	1.55	30.23	0.00	596.80	1–2	0.02	2.80	0.12	Small
	2	9.89	10	1.78			525.81	2–3	0.206	−1.82	−0.07	--
	3	9.62	10	1.87			481.48	3–1	0.00	5.49	0.19	Small
Identity	1	13.82	14	2.27	1.57	0.46	545.63	1–2	--	--	--	--
	2	13.68	14	2.17			515.25	2–3	--	--	--	--
	3	13.77	14	2.10			527.15	3–1	--	--	--	--
Self-focused	1	29.91	30	3.88	22.77	0.00	541.05	1–2	0.27	1.69	0.08	--
	2	29.46	30	3.72			584.63	2–3	0.06	−2.35	−0.09	--
	3	28.50	29	4.47			483.62	3–1	0.00	4.74	0.16	Small
Negativity	1	22.25	23	3.74	131.78	0.00	397.40	1–2	0.00	4.84	0.20	Small
	2	23.81	25	3.09			521.92	2–3	0.00	−4.85	−0.18	Small
	3	24.95	26	2.84			641.18	3–1	0.00	−11.47	−0.4	Small
Feeling “in between”	1	12.94	14	2.95	7.87	0.00	496.53	1–2	0.33	−1.60	−0.08	--
	2	13.35	14	2.67			537.26	2–3	1.00	0.74	0.03	--
	3	13.65	14	2.27			555.22	3–1	0.02	−2.78	−0.10	Small

**Table 5:** Means, Kruskal-Wallis, and *post-hoc* pairwise of the PERMA.

PERMA factors	Groups	Mean	Median	Standard deviation	Kruskal-Wallis			Post-hoc pairwise				
					H (2)	*p*	Rank	Peers	*p*	*z*	*r*	Effect
Positive emotions	1	21.20	22	4.31	421.88	0.00	765.69	1–2	0.00	7.80	0.32	Small
	2	20.02	20	4.77			564.50	2–3	0.00	−9.52	0.36	Small
	3	17.25	18	5.92			328.84	3–1	0.00	2.46	0.71	Medium
Engagement	1	22.68	23	4.74	153.86	0.00	674.66	1–2	0.00	5.02	0.21	Small
	2	20.64	20	4.77			545.09	2–3	0.00	5.45	0.21	Small
	3	17.79	18	5.92			410.40	3–1	0.00	12.38	0.43	Small
Relationships	1	22.78	23	5.19	195.08	0.00	685.68	1–2	0.00	4.57	0.19	Small
	2	20.41	21	5.93			567.70	2–3	0.00	7.16	0.27	Small
	3	16.14	17	7.06			390.59	3–1	0.00	13.82	0.48	Small
Meaning	1	21.63	23	5.75	234.62	0.00	710.58	1–2	0.00	6.54	0.27	Small
	2	17.70	18	6.96			519.59	2–3	0.00	6.39	0.24	Small
	3	13.78	14	7.17			398.75	3–1	0.00	15.31	0.53	Small
Accomplishments	1	20.92	22	4.78	206.38	0.00	705.18	1–2	0.00	7.20	0.30	Small
	2	17.51	18	5.22			519.59	2–3	0.00	4.88	0.19	Small
	3	14.98	15	5.99			398.75	3–1	0.00	14.35	0.50	Small
PERMA	1	109.21	113	19.35	359.03	0.00	705.64	1–2	0.00	7.66	0.38	Small
	2	93.61	94	21.80			552.96	2–3	0.00	8.34	−0.19	Small
	3	74.97	76	24.23			346.41	3–1	0.00	18.91	0.50	Medium

**Table 6:** Means, Kruskal-Wallis, and *post-hoc* pairwise of the CSs.

CS factors	Groups	Mean	Median	Standard deviation	Kruskal-Wallis			Post-hoc pairwise				
					H (2)	*p*	Rank	Pairs	*p*	*z*	*r*	Effect
Creativity	1	6.70	7	2.63	16.44	0.00	579.33	1–2	0.16	1.92	0.10	--
	2	6.27	6	2.47			529.98	2–3	0.41	−1.49	−0.06	--
	3	5.95	6	2.68			493.25	3–1	0.00	4.06	0.14	Small
Curiosity	1	8.99	9	2.47	17.58	0.00	578.50	1–2	0.34	1.58	0.08	--
	2	8.68	9	2.50			538.05	2–3	0.15	−1.96	−0.07	--
	3	8.21	9	2.74			489.95	3–1	0.00	4.17	0.15	Small
Open mindedness/openness to new ideas	1	8.90	9	2.27	2.94	0.23	544.97	1–2	--	--	--	--
	2	8.94	9	2.11			545.32	2–3	--	--	--	--
	3	8.70	9	2.21			512.92	3–1	--	--	--	--
Love of learning	1	8.05	8	2.70	54.55	0.00	613.60	1–2	0.03	2.59	0.11	Small
	2	7.11	7	2.65			547.15	2–3	0.00	−3.63	−0.14	Small
	3	6.59	6	2.86			457.71	3–1	0.00	7.33	0.25	Small
Perspective/wisdom	1	7.32	8	2.73	7.43	0.02	559.82	1–2	1.00	0.73	0.04	--
	2	7.18	8	2.51			541.15	2–3	0.37	−1.54	−0.06	--
	3	6.85	7	2.75			503.22	3–1	0.02	2.67	0.09	Small
Honesty	1	7.86	8	2.25	9.48	0.01	567.05	1–2	0.53	1.35	0.07	--
	2	7.29	8	2.34			532.45	2–3	0.64	−1.25	−0.05	--
	3	7.33	8	2.47			501.76	3–1	0.01	3.08	0.11	Small
Bravery	1	6.81	7	2.71	0.28	0.87	537.21	1–2	--	--	--	--
	2	6.50	7	2.79			525.94	2–3	--	--	--	--
	3	6.87	7	2.89			531.30	3–1	--	--	--	--
Persistence	1	8.14	8	2.65	94.49	0.00	639.24	1–2	0.00	3.32	0.14	Small
	2	7.32	8	2.89			553.80	2–3	0.00	−4.85	−0.18	Small
	3	6.07	6	3.12			434.16	3–1	0.00	9.64	0.33	Small
Enthusiasm/zest	1	7.29	8	2.61	306.80	0.00	727.74	1–2	0.00	6.23	0.33	Small
	2	5.63	6	2.81			567.59	2–3	0.00	−8.52	−0.32	Small
	3	3.47	3	2.69			357.36	3–1	0.00	17.40	0.60	Medium
Kindness/generosity	1	9.10	9	2.32	2.22	0.33	547.81	1–2	--	--	--	--
	2	8.92	9.5	2.52			516.41	2–3	--	--	--	--
	3	8.85	9	2.40			533.62	3–1	--	--	--	--
Love	1	7.70	8	2.41	73.80	0.00	628.85	1–2	0.00	3.30	0.14	Small
	2	6.70	7	2.42			447.07	2–3	0.00	−3.95	−0.15	Small
	3	6.14	6	2.73			544.24	3–1	0.00	8.56	0.30	Small
Social and emotional intelligence	1	7.34	8	2.51	4.48	0.11	557.08	1–2	--	--	--	--
	2	6.79	7	2.54			526.82	2–3	--	--	--	--
	3	6.92	7	2.82			512.41	3–1	--	--	--	--
Justice	1	9.07	9	2.11	2.75	0.25	542.66	1–2	--	--	--	--
	2	9.11	9	2.10			547.57	2–3	--	--	--	--
	3	8.90	9	2.09			513.65	3–1	--	--	--	--
Leadership	1	6.85	7	2.72	45.84	0.00	61.31	1–2	0.01	2.40	0.10	Small
	2	6.18	6	2.75			466.34	2–3	0.02	−2.78	−0.11	Small
	3	5.23	5	3.05			534.87	3–1	0.00	6.77	0.23	Small
Teamwork	1	8.05	8	2.40	32.28	0.00	595.94	1–2	0.27	1.70	0.09	--
	2	7.73	8	2.36			552.48	2–3	0.00	−3.40	−0.13	Small
	3	7.00	7	2.56			469.08	3–1	0.00	5.99	0.21	Small
Forgiveness	1	6.41	7	3.02	13.83	0.00	577.50	1–2	0.08	2.21	0.12	--
	2	5.87	5	3.15			52.68	2–3	1.00	−0.87	−0.03	--
	3	5.57	6	3.38			499.26	3–1	0.00	3.67	0.13	Small
Humility	1	8.45	9	2.24	1.20	0.55	535.98	1–2	--	--	--	--
	2	8.25	8	2.39			536.64	2–3	--	--	--	--
	3	8.44	9	2.29			511.46	3–1	--	--	--	--
Prudence	1	8.53	9	2.41	0.68	0.71	534.90	1–2	--	--	--	--
	2	8.62	9	2.25			541.50	2–3	--	--	--	--
	3	8.45	9	2.37			522.77	3–1	--	--	--	--
Self-regulation	1	7.07	7	2.61	24.94	0.00	587.38	1–2	0.20	1.83	0.10	--
	2	6.65	7	2.60			54.33	2–3	0.52	−2.38	−0.90	--
	3	6.17	6	2.73			481.81	3–1	0.00	4.97	0.17	Small
Appreciation	1	6.34	7	1.51	30.65	0.00	593.20	1–2	0.10	2.18	0.11	--
	2	6.05	6	1.37			539.58	2–3	0.03	−2.55	−0.10	Small
	3	5.55	6	1.74			477.57	3–1	0.00	5.52	0.19	Small
Gratitude	1	9.28	10	2.47	135.28	0.00	665.95	1–2	0.00	4.48	0.18	Small
	2	8.11	8	2.90			541.59	2–3	0.00	−4.98	−0.19	Small
	3	6.91	7	2.94			419.01	3–1	0.00	11.62	0.40	Small
Hope and optimism	1	8.81	9	2.66	176.79	0.00	608.24	1–2	0.00	4.96	0.20	Small
	2	7.45	8	3.15			453.12	2–3	0.00	−6.25	−0.24	Small
	3	5.70	6	3.34			565.16	3–1	0.00	13.23	0.46	Small
Humor	1	7.61	8	2.74	56.80	0.00	608.24	1–2	0.28	1.68	0.09	--
	2	7.19	6	2.89			453.12	2–3	0.00	−4.54	−0.17	Small
	3	6.07	6	3.02			565.16	3–1	0.00	13.23	0.46	Small
Spirituality	1	7.91	9	3.81	75.65	0.00	626.16	1–2	0.00	2.72	0.14	Small
	2	6.99	8	4.06			556.09	2–3	0.00	−4.57	−0.17	Small
	3	5.53	6	3.93			443.39	3–1	0.00	8.58	0.30	Small

## 4. Discussion

With regard to the levels of depression found, it was possible to verify a higher prevalence of severe/extremely severe depression. These results are contrary to those found by Frye and Liem (2011), who, in a sample of 2,944 emerging American adults, found that the highest prevalence of depression symptoms was in participants with lower depression levels. The study by Salmela-Aro et al. (2008) demonstrated that, among 297 Finnish emerging adults, the highest prevalence of depressive symptoms was for the moderate group.

The first hypothesis was confirmed in this study. The dimensions of emerging adulthood showed significant correlations with depression, however, with little amplitude. The identity factor was the only one of the emerging adulthood factors that did not show a significant correlation with depression and that did not reveal a difference in its means between the mental disorder groups, despite being considered its predictor, even in a less expressive way. These data may indicate that the consideration of the identity character was not relevant in the consideration of depression among the investigated sample.

Among all the emerging adulthood factors, the one that stands out the most is negativity, which was the one with the highest correlation amplitude, being a stronger prediction than identity, and which showed a small effect size among all symptomatology groups. This result is similar to the study by Othman and Jaafar (2022), where positive predictions of negativity and self-focus on depression were found. According to these authors, the problems and difficulties experienced in emerging adulthood can contribute to the experience of negative emotions in a generalized way, which can impact the consideration of this period as full of instabilities. The negative correlation of the Self-Focused factor and the positive correlation of the Feeling “In-Between” factor with depression may be an indicator of how self-beliefs are related to the prevalence of this disorder. According to Leme et al. (2021), self-efficacy explained 40% of the Self-Focused and 12% of the Feeling “In-Between” factors.

The correlations between the PERMA elements and depression presented moderate, negative associations, similar to what was found in other studies (Gander et al., 2016; Ryan et al., 2019; Kunjiapu and Kunasegaran, 2021; Wąsowicz et al., 2021). With regard to the predictions found among the PERMA factors, the Positive Emotions, Relationships and Meaning factors negatively predicted depression. However, the studies by Kern et al. (2015) and Falecki et al. (2018) indicated that all five elements of PERMA well-being could protect against negative emotions, depression and anxiety, and physical illness, as well as increase resilience and life satisfaction in adolescents and young adults. Finding positive emotions as negative predictors of depression is a result that can even be considered obvious (Wagner et al., 2019). However, this data should not be undervalued, on the contrary, it indicates the importance of promoting positive emotions or the capacity for emotional self-regulation among emerging adults, as indicated by Brewer et al. (2016) and Renna et al. (2018).

The significant negative correlations found in this study between character strengths and depression varied between fairly weak and moderate levels. Similarly, other studies have shown negative correlations between character strengths and depression (Duan and Wang, 2018; Casali et al., 2021; Feraco et al., 2021; Jabbari et al., 2021).

The second hypothesis was partially confirmed in this study. As expected, the negativity factor of emerging adulthood positively predicted depression. However, the identity factor within the same construct also showed a similar prediction. In light of these results, it can be inferred that the awareness of the identity assumed during this stage of life by emerging adults may be a contributing factor to experiencing higher levels of depression, especially when considering the more demanding and challenging aspects related to adult identity, such as life rules, obligations, and financial responsibilities, as pointed out by Galanaki and Sideridis (2018).

Although the size of the prediction of the Relationships factor was smaller than that of the Positive Emotions factor, it is necessary to emphasize the importance of this domain as a protective aspect for depression. Accordingly, reference should be made to the study by Pereira et al. (2018) which demonstrates the importance of the ability to engage socially and its protective relationships in emerging adulthood. The emotional benefits received through relationships with friends and family, which can be called social support, are of great relevance in this period of life (Pereira et al., 2018).

Likewise, the Meaning factor is important insofar as the more emerging adults experience this psychosocial resource, the lower their levels of depression. This result is similar to the study by Hoesni and Salleh (2022) who also found a negative predictive relationship between meaning in life and depression in a sample of emerging adults. According to the authors, the meaning of life is associated with a clear purpose and significance in life, with the satisfaction derived from this experience being capable of reducing undesirable emotional states.

Considering the predictions found by the analyses in this study, it can be seen that certain strengths act as a protective factor for depression, based on their negative predictions, while others appear as a risk factor for depression, due to their positive predictions. The only strength that negatively predicted depression was enthusiasm/zest, similar to the study by Bachik et al. (2021). Moreover, it is important to highlight the robustness of the negative prediction of enthusiasm/zest on depression, indicating it as a highly protective character strength against this mental disorder, as presented by Lam (2021).

The strengths of bravery, kindness and social/emotional intelligence presented positive predictions. Considering that character strengths are positive attributes that contribute to well-being, this result draws attention because it reveals itself from the opposite perspective. This result may lead to reflection on the overuse of some character strengths that can harm the individual’s mental health, as explained by Barros et al. (2022).

Similarly, Tehranchi et al. (2018) selected seven character strengths (hope, open mindedness, emotional intelligence, gratitude, forgiveness, spirituality, and enthusiasm) in assessing the etiology of depressive symptoms in a sample of 200 Iranians aged between 18 and 50 years. The results showed that these strengths had an influence on the direct relationships of dysfunctional attitudes and happiness with depression, that is, the seven forces had an unexpected effect on depression.

The third hypothesis was partially confirmed in this study. To date, no investigations have been found that compared emerging adulthood dimensions and PERMA levels among different scores of depressive symptoms. The negativity factor of emerging adulthood was the one that showed the greatest differences in its levels among the different symptomatology groups, as expected for this element.

Comparisons between the groups in this sample showed that higher levels of depression equated to lower scores in the elements that comprise the PERMA. The results presented here may serve as important points of reflection on the relevance of promoting PERMA well-being attributes among emerging adults, especially those who have higher levels of depressive symptoms and lower PERMA levels, as suggested by Gander et al. (2016).

As with the other elements, no studies were found that evaluated character strength scores among groups with different levels of depression. Similar results were found by Anjum and Amjad (2016), which explain the differences in the levels of character strengths in relation to the groups that received university counseling, those who needed to participate in this service and those who reported not depending on this support. These groups could be associated with the division of symptoms in this work. The vast majority of character strengths demonstrated a progressive increase in their levels as the levels of depression increased, with the exception of a few elements that oscillated their scores across symptomatology groups. This finding prompts reflection on the effects of the utilization of certain character strengths on individuals’ mental health.

Emerging adulthood is a phase of development accompanied by changes and challenging demands. The aim of this study was to verify how factors of emerging adulthood, PERMA and character strengths correlated and predicted depression and differed in relation to the levels of this disorder in a sample of emerging adults. The results demonstrated that some factors of the independent variables were correlated and predicted depression positively and negatively and that there were significant differences in several levels of these attributes. In this way, it was possible to advance a little further in the understanding of the relationships between the factors of the dependent variables and depression in this phase of human development.

The results indicate the importance of considering the characteristics emphasized by the five factors of emerging adulthood in depression, with the exception of identity in this study, showing that the conditions typical of this phase of life can favor and also hinder the development of depression. In this way, the specificities of emerging adulthood must be taken into account, in order to offer this population ways to enhance the experiences that refer to the possibilities and self-focus factors. In the same way, the development of personal resources that can lead them to overcome difficulties in experiences related to the negativity and ambivalence factors should be encouraged.

Regarding the PERMA, its value in relation to depressive symptoms can be corroborated, with emphasis on the Positive Emotions, Relationships and Meaning elements, which, in addition to being correlated, were negative predictors of depression. The means of these elements presented the greatest magnitude of differences among the depression groups in relation to the other factors investigated in this study.

Considering the numerous character strength factors, it was possible to perceive that, in relation to depression, the strengths can be considered beneficial, emphasizing the role of enthusiasm that presented itself in a negatively associated and predictive way, and that together with love of learning, persistence, love and the strengths that make up the virtue of transcendence (appreciation of beauty, gratitude, hope and optimism, humor and spirituality) showed significant differences among almost all groups.

The results of this study have significant theoretical and practical implications for Positive Psychology, particularly regarding the relationship between the elements of emerging adulthood, the PERMA well-being model, character strengths, and depression. The theoretical implications are that these findings contribute to understanding the factors that influence the development of depression during emerging adulthood, confirm the importance of these elements for mental well-being and emphasizes the need to promote positive emotions, healthy relationships, and a sense of purpose and meaning in emerging adults lives and indicate that the balanced use of character strengths can be crucial for mental health and that increasing awareness of the overuse of certain strengths may be important for preventing depression.

These data related to the elements of these two significant Positive Psychology constructs that emphasize the importance of promoting them in populations of emerging adults, along the emerging adulthood factors. This study can serve as a valuable resource in the construction Positive Psychology Interventions by identifying the factors that should be prioritized in the creation and implementation of these programs allowing professionals to develop more effective and personalized intervention programs aimed at promoting mental well-being and preventing depression in this population.

In addition, he results also have implications in the realm of public policies, including healthcare and education. By highlighting the importance of Positive Psychology elements in preventing depression and promoting mental well-being, these results can support the implementation of specific actions and programs targeting emerging adults. This may include integrating mental health promotion strategies into healthcare policies, as well as incorporating content related to well-being and emotional resilience into educational curricula.

Despite its relevance, this study has methodological limitations such as the non-normality of the sample that did not allow the performance of ANOVA or MANOVA tests which would have allowed a deeper understanding of the associations between the variables. The sample of participants also does not represent the Brazilian population of emerging adults and its sociodemographic diversity, thus preventing the broad generalization of the results. In view of these limitations and based on the questions that arose during the development of this study, we suggest investigations that verify the associations of the variables through even more robust statistical analyses and that include other variables that were not considered in this study. This could provide longitudinal results that explain the development of both depression and individuals’ positive resources over time.

## Data availability statement

The raw data supporting the conclusions of this article will be made available by the authors, without undue reservation.

## Ethics statement

The studies involving humans were approved by the Comitê de Ética da Universidade Salgado de Oliveira. The studies were conducted in accordance with the local legislation and institutional requirements. The participants provided their written informed consent to participate in this study.

## Author contributions

AS and AB: conceptualization, methodology, visualization, and project administration. AB: formal analysis, investigation, writing – original draft preparation, and resources. AS: writing – review and editing, funding acquisition, and supervision. All authors contributed to the article and approved the submitted version.

## Funding

This work was financed by Coordenação de Aperfeiçoamento de Pessoal de Nível Superior and Fundação Carlos Chagas Filho de Amparo à Pesquisa do Estado do Rio de Janeiro.

## Conflict of interest

The authors declare that the research was conducted in the absence of any commercial or financial relationships that could be construed as a potential conflict of interest.

## Publisher’s note

All claims expressed in this article are solely those of the authors and do not necessarily represent those of their affiliated organizations, or those of the publisher, the editors and the reviewers. Any product that may be evaluated in this article, or claim that may be made by its manufacturer, is not guaranteed or endorsed by the publisher.
